# Chemical profiling and cytotoxic potential of the *n*-butanol fraction of *Tamarix nilotica* flowers

**DOI:** 10.1186/s12906-023-03989-8

**Published:** 2023-05-24

**Authors:** Marwa A. A. Fayed, Riham O. Bakr, Nermeen Yosri, Shaden A. M. Khalifa, Hesham R. El-Seedi, Dalia I. Hamdan, Mohamed S. Refaey

**Affiliations:** 1 Department of Pharmacognosy, Faculty of Pharmacy, University of Sadat City, Sadat City, 32897 Egypt; 2 Department of Pharmacognosy, Faculty of Pharmacy, October University for Modern Sciences and Arts (MSA), Giza, Egypt; 3 Chemistry Department of Medicinal and Aromatic Plants, Research Institute of Medicinal and Aromatic Plants (RIMAP), Beni-Suef University, Beni-Suef, 62514 Egypt; 4 Department of Molecular Biosciences, The Wenner-Gren Institute, Stockholm University, S-106 91 Stockholm, Sweden; 5 International Joint Research Laboratory of Intelligent Agriculture and Agri-Products Processing, Jiangsu Education Department, Jiangsu University, Nanjing, 210024 China; 6 Pharmacognosy Group, Department of Pharmaceutical Biosciences, Uppsala University, Biomedical Centre, P.O. Box 591, 751 24 Uppsala, SE Sweden; 7 International Research Center for Food Nutrition and Safety, Jiangsu University, Zhenjiang, 212013 China; 8 Department of Chemistry, Faculty of Science, Menoufia University, Shebin El-Koom, 32512 Egypt; 9 Department of Pharmacognosy and Natural Products, Faculty of Pharmacy, Menoufia University, Shebin El-Koom, 32511 Egypt

**Keywords:** *Tamarix nilotica* flowers, LC–LTQ–MS–MS, ^1^H-NMR, Cytotoxicity, MCF-7, Huh-7

## Abstract

**Background:**

Cancer represents one of the biggest healthcare issues confronting humans and one of the big challenges for scientists in trials to dig into our nature for new remedies or to develop old ones with fewer side effects. Halophytes are widely distributed worldwide in areas of harsh conditions in dunes, and inland deserts, where, to cope with those conditions they synthesize important secondary metabolites highly valued in the medical field. Several *Tamarix* species are halophytic including *T.nilotica* which is native to Egypt, with a long history in its tradition, found in its papyri and in folk medicine to treat various ailments.

**Methods:**

LC–LTQ–MS–MS analysis and ^1^H-NMR were used to identify the main phytoconstituents in the *n*- butanol fraction of *T.nilotica* flowers. The extract was tested  in vitro for its cytotoxic effect against breast (MCF-7) and liver cell carcinoma (Huh-7) using SRB assay.

**Results:**

*T.nilotica n*-butanol fraction of the flowers was found to be rich in phenolic content, where, LC–LTQ–MS–MS allowed the tentative identification of thirty-nine metabolites, based on the exact mass, the observed spectra fragmentation patterns, and the literature data, varying between tannins, phenolic acids, and flavonoids. ^1^H-NMR confirmed the classes tentatively identified. The in-vitro evaluation of the *n*-butanol fraction showed lower activity on MCF-7 cell lines with IC_50_ > 100 µg/mL, while the higher promising effect was against Huh-7 cell lines with an IC_50_= 37 µg/mL.

**Conclusion:**

Our study suggested that *T.nilotica* flowers' *n*-butanol fraction is representing a promising cytotoxic candidate against liver cell carcinoma having potential phytoconstituents with variable targets and signaling pathways.

## Introduction

All over the world, cancer ranks as a primary cause of mortality and a significant roadblock to raising life expectancy [1, 2]. According to World Health Organization (WHO) estimations for 2022, globally cancer represented the cause of death for 16% before the age of 70 [3]. Hepatocellular carcinoma is the predominant primary cancer in most countries and the fourth most prevalent cancer across the globe [4, 5] besides being the third most lethal cancer-associated mortality in the world [6].

Additionally, breast cancer represents the first-leading cause of death for women, almost 2.3 million women received a breast cancer diagnosis in the world in 2020, and 685,000 of them passed away. Somewhere in the globe, a woman receives a breast cancer diagnosis every 14 s [6, 7]. The main regimen of treatment of various forms of cancer is to stop unregulated cell growth which can be achieved by using cytotoxic drug medications. The effect of these drugs can be estimated by using cell-based in vitro assays to measure the degree of tissue-level cell damage [8].

However, the use of conventional chemotherapeutic agents has been associated with a wide range of side effects and toxicities; therefore, new approaches for the prevention and cure of cancer represent a great challenge for researchers [9]. One of the most crucial methods for treating particular types of cancer is the discovery of natural anti-cancer medications, which requires constant monitoring of various sources such as marine animals, terrestrial plants, and seaweeds [10].

There are more than 60 species of halophyte plants in the genus *Tamarix*  belonging to the Tamaricaceae family, which are cultivated in almost every region of the world under the common names “Tamarisk” and “salt cedar” [11, 12]. It has a variety of therapeutic uses in conventional medicine [11]. Due to the plant’s astringent and cleaning properties on internal organs, which were attributed to its bitter taste, it was known to have a chilly and dry nature [11]. Certain *Tamarix* species are recommended as mild laxatives, anti-tussive, antipyretics, and tonics for the liver and spleen [11, 13]. Some species are used to treat leucorrhea and uterine bleeding because they have anti-inflammatory and wound-healing characteristics [14]. It can be applied topically to treat skin conditions like eczema and anal fissure [13]. Biological studies have demonstrated that some *Tamarix* species can be used as anti-Alzheimer [15], anti-diabetic [16], anti-hyperlipidemic [17], anti-inflammatory [18, 19], antimicrobial [20, 21], antinociceptive [22], antioxidant [23], anti-coagulation [24], anti-rheumatoid [25], cytotoxicity [26], hepatoprotective [27] and wound healing [28] activities. *Tamarix* is represented in Egypt with two indigenous species which are *T. aphylla* (L.) H. Karst and *T. nilotica* (Ehrenb.) Bunge. *T. nilotica* is a rich source of polyphenolics including hydrolyzable tannins, sulfated and non-sulfated flavonoids, and phenylpropanoids [29, 30]. *T. nilotica* extracts have demonstrated antioxidant, antiangiogenic, cytotoxic, hepatoprotective, antifibrotic, antidiabetic, and antimicrobial activities in relation to their phenolic contents [29–31]. Although both species are indigenous in Egypt, many studies targeted *T. aphylla* which was mentioned for comparison to *T. nilotica* [16, 20, 22, 28, 32–35]. Besides, *T. nilotica* was the one easily available for us to carry on with this study.

In the previous published studies, *T. nilotica* received much attention in studying its cytotoxic activity. Various studies reported the effect of leaves, methanolic flower extracts on different cell lines including lung (A-549), liver (Huh-7), colon (HCT-116), and breast (MCF-7) cancer cell lines [36–38]. *T. nilotica* flower extract reported to exhibit hepatoprotective and antioxidant activities [38]. However, there are no studies concerning the cytotoxic activities of the *n*-butanol fraction of *T. nilotica* flower.

The present work aimed to investigate the possible cytotoxic activity of the *n*-butanol extract of *T. nilotica* flowers against liver (Huh-7) and breast (MCF-7) cell carcinoma while performing an in-depth phytochemical analysis of the same extract *n*-butanol extract using LC-MS/MS analysis to relate the activity to the extract’s metabolites.

## Methods

### Statement

All experiments and methods including the collection of the plant were performed following the relevant national, and international guidelines and legislation of the Faculty of Pharmacy, University of Sadat City, Sadat City, Egypt.

### Extraction and Isolation

The air-dried flowers of *T. nilotica* (Ehrenb.) Bunge (1 kg) was exhaustively extracted with 80% methanol; excess solvent was removed using a rotary evaporator. The crude aqueous methanolic extract was further fractionated using solvents of different polarity viz., *n*-hexane, dichloromethane, *n*-butanol, and water. The fractions were dried under vacuum to give their corresponding weights of 30 gm, 25 gm 15 gm, and 45 gm, respectively. All fractions were stored at -20 °C till further analysis [39].

### LC–LTQ–MS–MS analysis

The *n-* butanol extract was analyzed and processed using LC–MS–MS. A Shimadzu LC-10 HPLC with a Grace Vydac Everest Narrowbore C-18 column (100 mm × 2.1 mm i.d., 5 μm, 300 Å). An LC–MS, connected to an LTQ Linear Ion Trap MS (Thermo Finnigan, San Jose, CA) was utilized with a mass range of 100–2000 m/z. A 2 µL sample was injected using an autosampler. A 35 min method was used as follows: 5 min isocratic run using 5% acetonitrile (Acn) and 0.05% formic acid (FA), then a gradient was run for 25 min until 95% AcN 0.05% FA. Finally, there was 5 min of conditioning the column with 5% AcN and 0.05% FA. The data were processed and analyzed using foundation 3.1_Xcalibur_3.1.6610 as well as MZmine3. Furthermore, the raw data files were converted to mzXML format using MSConvert from the ProteoWizard suite [40]. The molecular network was created using the Global Natural Products Social Molecular Networking (GNPS) online workflow. The spectra in the network were then searched against the GNPS spectral libraries and published data [41, 42].

Using the GNPS dataset, the raw MS file was analyzed. By analyzing the similarity between the fragmentation pattern from the raw mass spectrum and the GNPS library, GNPS assists in the identification and discovery of metabolites. Other installed programs, including MSConvert (https://proteowizard.sourceforge.io/), File Zilla (https://filezilla-project.org/), and Cytoscape version 3.5.1(https://cytoscape.org/), were used to operate with GNPS at the following link (https://gnps.ucsd.edu/) [43, 44].

### ^1^H-NMR analysis

^1^H-NMR spectra were recorded at 298 K on a Bruker 600 MHz (TCI CRPHe TR-^1^H and ^19^F/^13^C/^15^N 5 mm-EZ CryoProbe) spectrometer. Chemical shifts were referenced to the solvent peak for CH_3_OD at δ_H_ 3.3100 ppm [44, 45].

### Cytotoxic evaluation of the *n*-butanol fraction of *T. nilotica* flowers

#### Cell cultures

Breast adenocarcinoma cell lines (MCF-7) and hepatocyte-derived cellular carcinoma cell lines, human liver (Huh-7) was obtained from Nawah Scientific Inc., (Mokatam, Cairo, Egypt). Cells were maintained in DMEM media supplemented with 100 mg/mL of streptomycin, 100 units/mL of penicillin, and 10% of heat-inactivated fetal bovine serum in humidified, 5% (v/v) CO_2_ atmosphere at 37 °C [46].

### Cell cytotoxicity

Cell viability was assessed by sulforhodamine B (SRB) assay on two cancer cell lines [47, 48], the human liver cancer cell line (Huh-7) and the breast cancer cell line (MCF-7). Aliquots of 100 µL cell suspension (5 × 10^3^^ cells) were in 96-well plates and incubated in complete media for 24 h. Cells were treated with another aliquot of 100 µL media containing the *n*-butanol *T. nilotica* flower extract at two different concentrations (10 and 100 µg/ml). After 72 h, cells were fixed by replacing media with 150 µL of 10% TCA and incubated at 4 °C for 1 h. The TCA solution was removed, and the cells were washed 5 times with distilled water. Aliquots of 70 µL SRB solution (0.4% w/v) were added and incubated in a dark place at room temperature for 10 min. Plates were washed 3 times with 1% acetic acid and allowed to air-dry overnight. Then, 150 µL of TRIS (10 mM) was added to dissolve the protein-bound SRB stain; the absorbance was measured at 540 nm using a BMG LABTECH- FLUOstar Omega microplate reader (Ortenberg, Germany) [49]. The cell morphological analysis was carried out according to M. Roy et al. 2017 [50].

### Statistical analysis

Statistical analysis of the data was performed using one-way ANOVA, followed by Tukey’s multiple range tests for post hoc comparisons (GraphPad Prism, version 8.4.2). All the data are presented as the means of 3 determinations ± SE [51].

## Results

### Metabolic profiling of the *n*-butanol fraction of *T. nilotica* flowers using LC–LTQ–MS–MS analysis in positive mode

**Fig. 1:**
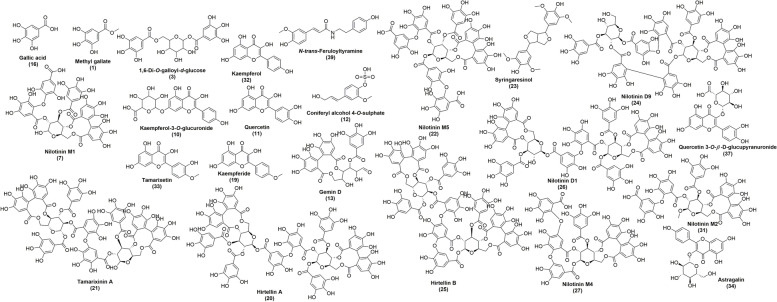
Chemical structures of the tentatively identified compounds in the *n*-butanol fraction of *T. nilotica* flowers numbered according to compounds listed in Table 1

Based on the exact mass, the observed spectra fragmentation patterns, and literature data, the structural characterizations of chemical composition in the *n*-butanol fraction of the *T. nilotica* flowers were accomplished. Using MS/MS fragmentation pattern, 39 compounds from various classes of secondary metabolites were identified. The detected compounds’ structures were presented in (Fig. 1). Molecular ion, retention time, and MS/MS data of identified compounds were provided in (Table 1).

**Table 1:** Metabolites tentatively identified from the *n*-butanol fraction of *T. nilotica* flowers using LC–LTQ–MS–MS analysis in positive mode

No.	Identification	Molecular formula	Exact mass	R_t_ (min)	m/z	MS/MS fragments	Ref.
					**(+ ve)**	**(+ ve)**	
**1**	Methyl gallate	C_8_H_8_O_5_	184.0371	0.64	184.9999	125.9427-141.9137	[52]
**2**	Morphinan-4,6-diol, N-formyl-6-acetate(ester)	C_19_H_23_NO_4_	329.16271	2.31	330.1706	260.1651	[53]
**3**	1,6-Di-*O*-galloyl-*d*-glucose (nilocitin)	C_20_H_20_O_14_	484.0853	2.42	485.0025	171.0516-315.0885- 333.0927	[30, 54]
**4**	Hispidulin	C_16_H_12_O_6_	300.06339	7.08	300.9978	287.0618, 271.0781	[55]
**5**	Methyl gallate methyl ether	C_9_H_10_O_5_	198.05282	7.53	199.0607	183.2035, 182.1017, 168.1108, 167.1539	[30]
**6**	Luteolin	C_15_H_10_O_6_	286.0477	8.65	286.9991	259.0632, 153.0582, 137.087	[34]
**7**	Nilotinin M1	C_41_H_30_O_27_	954.0974	9.67	955.0017	483.0583-321.0531	[56]
**8**	5-Hydroxy-3,7, 4’ -trimethoxyflavone	C_18_H_16_O_6_	328.09469	10.68	329.1040	314.9954., 301.1168, 286.0685	[57]
**9**	Methylquercetin hexoside (tamarixetin-3-*O*-hexoside)	C_22_H_22_O_12_	478.1111	11.13	478.9998	316.9950- 302.0865	[30]
**10**	Kaempferol-3-*O*-glucuronide	C_21_H_18_O_12_	462.0798	11.52	463.001	287.0548-259.0584	[58]
**11**	Quercetin	C_15_H_10_O_7_	302.0426	12.64	302.9995	181.0502- 274.9857- 153.0431	[54]
**12**	Coniferyl alcohol 4-*O*-sulphate	C_10_H_12_O_6_S	260.0354	13.09	260.9994	231.0484- 181.0399	[59]
**13**	Gemin D	C_27_H_22_O_18_	634.0806	14.04	634.9988	483.1707-321.1121-303.0972	[60]
**14**	Pilloin	C_17_H_14_O_6_	314.07904	14.44	315.0879	301.1345, 287.1154	[53]
**15**	Remurin A	C_48_H_34_O_31_	1106.10842	15.82	1107.1155	650.3398- 498.4456-346.522	[61]
**16**	Gallic acid	C_7_H_6_O_5_	170.0215	17.13	171.0005	126.936	[30]
**17**	Ferulic acid	C_10_H_10_O_4_	194.05791	17.29	195.06574	179.1750, 150.1777, 135.0983	[30]
**18**	Caffeic acid	C_9_H_8_O_4_	180.04226	21.54	181.0008	163.0144	[34]
**19**	4’-Methyl kaempferol (Kaempferide)	C_16_H_12_O_6_	300.0633	22.75	301.0015	286.0854-273.0591	[30, 62]
**20**	Hirtellin A	C_82_H_58_O_52_	1874.1894	23.32	1874.9932	1722.399-1416.418-1263.593	[56]
**21**	Tamarixinin A	C_75_H_52_O_48_	1720.1628	25.19	1720.9955	1569.374-1416.329-483.5862-320.9474	[63]
**22**	Nilotinin M5	C_55_H_38_O_36_	1274.1142	25.59	1274.9998	1123.457-971.7501-819.6556-483.5314	[64]
**23**	Syringaresinol	C_22_H_26_O_8_	418.1627	26.49	418.9981	329.5263-373.5963-389.6274	[65]
**24**	Nilotinin D9	C_68_H_50_O_44_	1570.1675	26.61	1570.9984	1419.444-1266.923	[66]
**25**	Hirtellin B	C_82_H_56_O_52_	1872.1737	27.98	1872.9917	1721.137-1416.851	[67]
**26**	Nilotinin D1	C_75_H_54_O_48_	1722.1784	28.27	1723.0042	1570.922-1418.1300-1265.0900	[29]
**27**	Nilotinin M4	C_48_H_32_O_31_	1104.0927	28.49	1105.0016	953.718-801.6526-483.6066	[68]
**28**	1,2,6-Tri-*O*-galloyl-*β*-*D*-glucose	C_27_H_24_O_18_	636.0962	29.78	636.9999	465.9667-423.9695-483.8437	[69]
**29**	Kaempferol dimethyl ether sulphate	C_17_H_14_O_9_S	394.0358	30.28	395.0009	315.0898- 300.1266- 285.0565	[30, 54]
**30**	Methylquercetin-sulphate (tamarixetin sulphate)	C_16_H_12_O_10_S	396.0151	31.57	397.0016	317.0424- 302.349- 219.0595	[30, 32]
**31**	Nilotinin M2	C_42_H_32_O_27_	968.1131	31.85	968.9999	954.2317-483.8324-321.0566	[70]
**32**	Kaempferol	C_15_H_10_O_6_	286.0477	32.46	286.9988	241.148-145.0603	[32]
**33**	4’-*O*-Methylquercetin (Tamarixetin)	C_16_H_12_O_7_	316.0583	32.85	316.9999	302.0346-195.0663	[30, 62]
**34**	Kaempferol-3-*O*-glucoside (Astragalin)	C_21_H_20_O_11_	448.1005	33.3	449.0009	449.0009-328.0134-287.0151	[71]
**35**	Kaempferol methyl ether sulphate	C_16_H_12_O_9_S	380.0202	33.75	380.9984	301.0015- 286.0854	[30, 59]
**36**	5,7,4’-trihydroxy-3’-methoxylflavone	C_16_H_12_O_6_	300.0633	33.75	301.0015	286.0854-153.0438-135.0147	[72]
**37**	Quercetin-3-*O*-*β* -*D*-glucupyranuronide	C_21_H_18_O_13_	478.0747	33.86	479.0021	303.1093-178.0701	[72, 73]
**38**	N-*trans*-Feruloyltyramine	C_18_H_19_NO_4_	313.1314	34.09	314.0005	299.1171-180.0647-358.056 (M + HCOO)^+^	[74]
**39**	Ferulic acid sulfate derivative	C_10_H_10_O_7_S	274.0147	34.37	274.999	230.0479-195.0351-200.0469	[75]

### LC–LTQ–MS–MS analysis of the *n*-butanol fraction of *T. nilotica* flowers using GNPS-Aided annotation

**Fig. 2:**
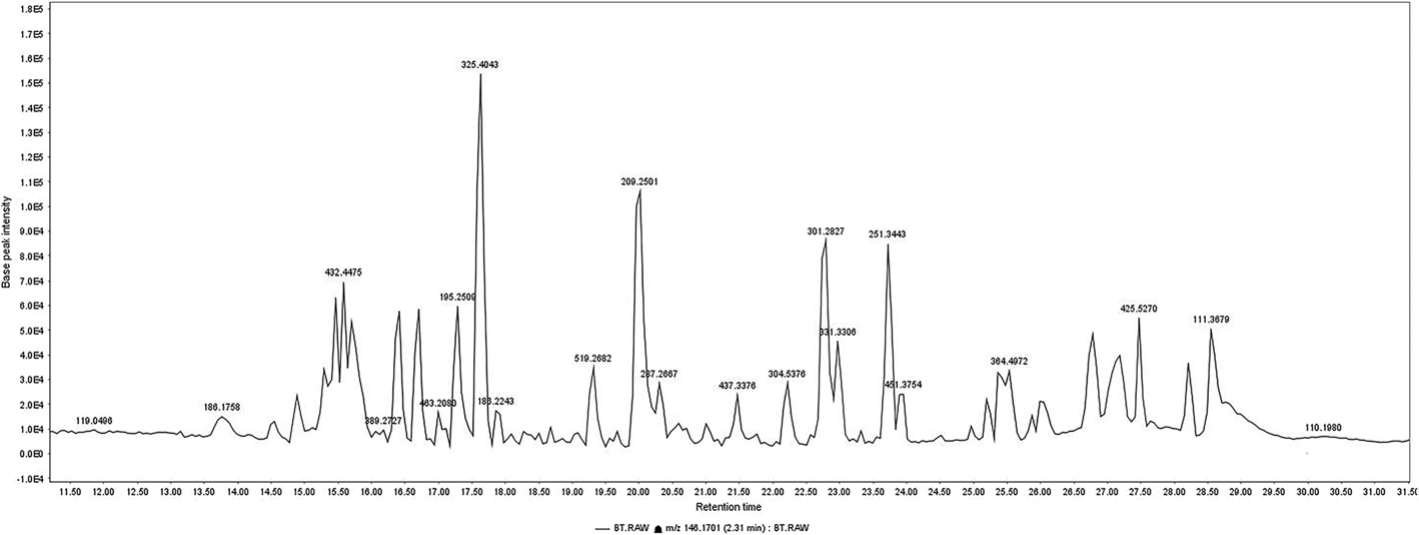
LTQ-LC-MS-MS chromatogram of the *n*- butanol fraction of *T. nilotica* flowers

**Fig. 3:**
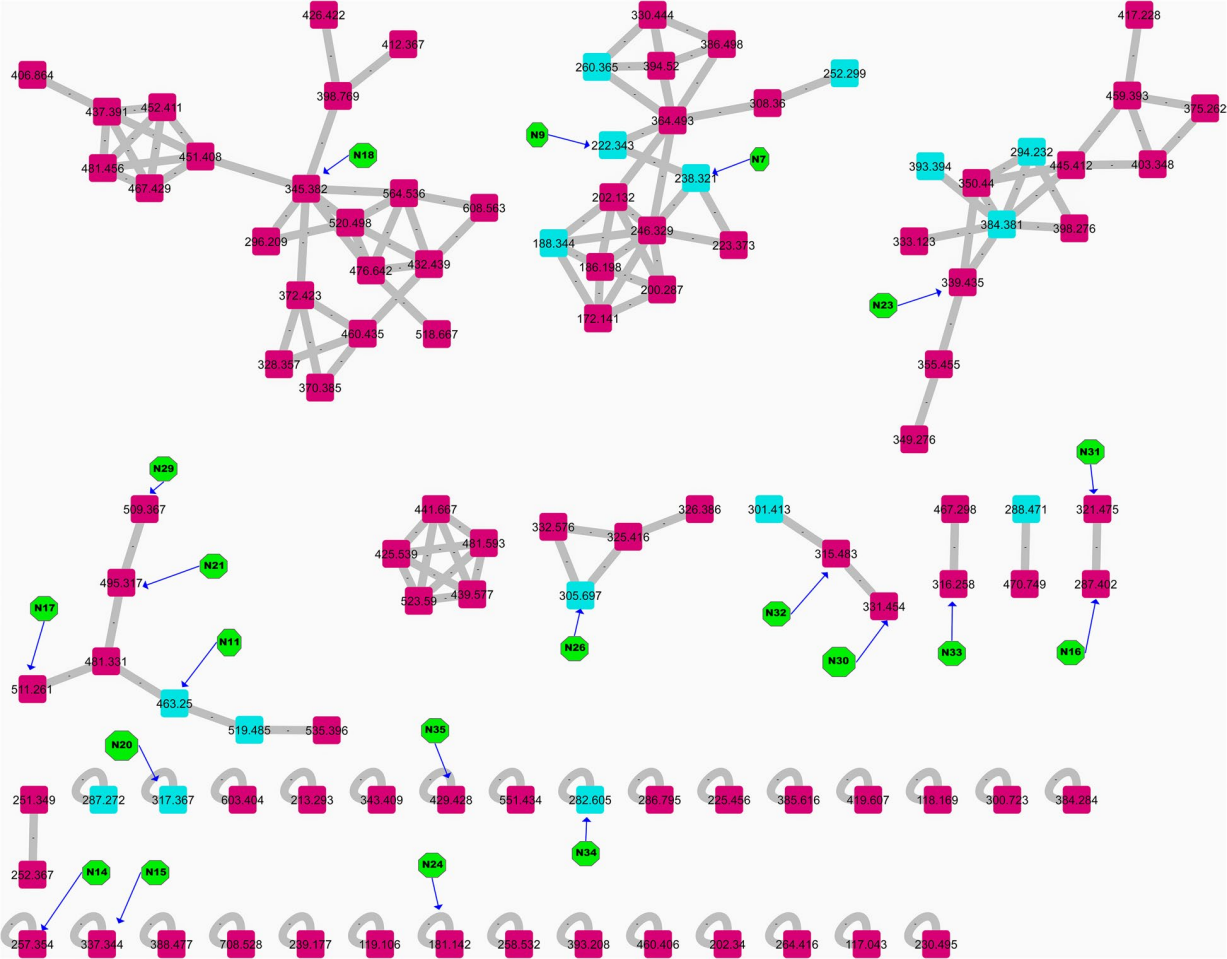
Molecular network (showing clusters of metabolites of interest) based on tandem mass spectrometry data in the positive ionization mode of the *n*-butanol fraction of *T. nilotica* flowers. Twenty metabolites have been identified as labeled in Fig. 3, green color indicating the number of compounds in Table 2, light blue nodes are compounds identified using GNPS databases, while the identified compounds using fragmentation matching have the pink color

Metabolite profiling of the *n*-butanol fraction of *T. nilotica* flowers *via* GNPS based on tandem mass spectrometry data as well as a dictionary of natural products yielded the annotation of 35 metabolites (N1—N35); mainly flavonoids, phenolics, and fatty acids; respectively (Figs. 1 and 2; Table 2). Flavonoids were annotated by observing the common fragments of retro dials-alder reaction indicated at *m/z* 153, 152, 135 depending on structure as in N11, 15, 16, 17, 18, etc. Additionally, common fragments such as [M-18 Da] denoting loss of H_2_O molecule, [M-28 Da] denoting the loss of CO, [M + H-42]^+^ corresponding to C_2_H_2_O loss, besides [M + H-46]^+^, as in quercetin, kaempferol, and myricetin derivatives. A common fragment in *O*-methylated flavonoids is [M + H-15]^+^ formed by loss of methyl radical, as shown in N10 (Kaempferide-*O*-hexoside), N21 (Kaempferide-*O*-hexoside derivative), N28 (kaempferide), N20 **(**tamarixetin), N32 (kaempferol 4’,7-dimethyl ether), N30 (quercetin- dimethyl ether) and N18 (herbacetin-trimethyl ether). Flavanones were annotated in the form of dihydro derivatives of flavonols as presented in N26 (*m/z* 305) tentatively identified as dihydro-quercetin, N31 (*m/z* 321) identified as dihydromyricetin. Phenolic acids i.e., N5, N12, N13, and N24 were previously reported in *Tamarix* species. GNPS databases also aided in identifying N7, N9, N14, N25, and N34, besides kaempferol derivatives as well (Fig. 3).

**Table 2:** Metabolites identified from the *n*-butanol fraction of *T. nilotica* flowers based on NMR and GNP analysis. **No**. = numbers of identified metabolites, **R**_**t**_= retention time in mins, **MF** = molecular formula, **ID** = name of identified compounds, **Ref**. = references of identified compounds

No.	R_t_	[M + H]^+^	MF	Fragmentation	ID	Ref.
**1.**	2.27	146.09	C_6_H_11_NO_3_	127.92, 99.91	Hydroxyproline; *N*-Me	[76]
**2.**	2.35	277.19	C_13_H_8_O_7_	259.04, 185.00, 144.75, 114.94	Urolithin M5	[77]
**3.**	3.11	132.19	C_5_H_9_NO_3_	113.94, 99.92, 85.93	Hydroxyproline	[76]
**4.**	3.14	333.11	C_18_H_20_O_6_	315.00, 297.08, 252.98, 240.06	Tamarixoic acid	[35]
**5.**	5.38	166.07	C_9_H_8_O_3_	148.98, 119.9361	Coumaric acid	[34]
**6.**	14.90	160.19	C_7_H_13_NO_3_	142.99, 114.00, 86.91	Hydroxyproline; *N,N*-Di-Me/ betaine	[76]
**7.**	15.86	238.32	C_13_H_19_NO_3_	221.02, 135.97	Tyrosine butyl ester	GNPS
**8.**	16.11	635.43	C_27_H_22_O_18_	617.02, 465.08, 302.96	Gemin D	[60]
**9.**	16.72	222.34	C_13_H_19_NO_2_	204.97, 165.93, 119.98	Phenylalanine, butyl ester	GNPS
**10.**	17.03	464.25	C_21_H_22_O_12_	446.13, 301.00, 287.98	Kaempferide-*O*-hexoside	[78]
**11.**	17.07	463.28	C_22_H_22_O_12_	286.97, 150.98	Kaempferol-*O*-glucuronide	[79]
**12.**	17.21	171.33	C_7_H_6_O_5_	163.77, 152.97, 122.88	Gallic acid	[80]
**13.**	17.30	195.24	C_10_H_10_O_4_	177.05	Ferulic acid	[80]
**14.**	18.87	257.31	C_16_H_32_O_2_	239.02, 174.9, 92.92	Palmitic acid	GNPS
**15.**	18.93	337.35	-	319.12, 301.144, 283.20, 259.17, 149.05	Myricetin derivative	[81]
**16.**	19.00	287.62	C_15_H_10_O_6_	269.01, 240.96, 213.06, 188.02, 152.97	Kaempferol	[72]
**17.**	19.54	511.27	-	493.07, 387.08, 303.04, 317.02, 152.93	Tamaridone-*O*-hexoside derivative	[82]
**18.**	19.83	345.49	C_18_H_16_O_7_	237.17, 289.00, 270.90, 242.97, 152.95	Dihydroxy-trimethoxyflavone/ Herbacetin-trimethyl ether	[83]
**19.**	19.97	209.28	C_10_H_8_O_5_	177.04	Trihydroxy-methylcoumarin.	[84]
**20.**	20.23	317.40	C_16_H_12_O_7_	301.96, 270.98, 164.98	*O*-Methylquercetin (Tamarixetin)	[78]
**21.**	20.81	495.31	-	477.08, 463.05, 300.99, 286.98, 152.99	Kaempferide-*O*-hexoside derivative	[85]
**22.**	21.03	496.37	-	478.08, 301.98, 153.04	quercetin derivative	[85]
**23.**	21.36	339.47	C_15_H_14_O_7_S	321.19, 303.22, 285.13, 251.15, 207.12	Trihydroxyflavan 7-Sulfate	[86]
**24.**	21.78	181.27	C_9_H_8_O_4_	162.98, 134.96	Caffeic acid	[34]
**25.**	21.79	283.36	C_18_H_34_O_2_	265.13, 248.13	Oleic acid	GNPS
**26.**	22.20	305.56	C_15_H_12_O_7_	287.08, 269.11, 259.10, 213.15	Dihydro-quercetin	[87]
**27.**	22.23	302.30	C_15_H_10_O_7_	286.97, 272.99, 228.09, 152.93, 138.89	Quercetin	[72]
**28.**	22.75	301.41	C_16_H_12_O_6_	285.97, 271.98, 227.01, 18,806, 152.90, 138.91	Kaempferide	[78]
**29.**	22.78	509.39	-	477.08, 315.00, 301.00, 166.95	Kaempferol 4’,7-dimethyl ether-*O*-hexoside derivative	[88]
**30.**	22.92	331.41	C_17_H_14_O_7_	315.99, 299.02, 275.03, 178.95, 152.96	Tamaridone/ quercetin- dimethyl ether	[34]
**31.**	24.60	321.46	C_15_H_12_O_8_	303.16, 285.19, 247.03, 222.05, 174.10	Dihydromyricetin	[89]
**32.**	25.38	315.26	C_17_H_14_O_6_	300.00, 285.99, 272.02, 152.90	Kaempferol 4’,7-dimethyl ether	[34]
**33.**	25.39	316.41		301.01, 287.12, 273.02, 152.97	Quercetin derivative	[90]
**34.**	27.00	282.28	C_18_H_35_NO	265.13, 247.13	Octadecenamide	GNPS
**35.**	28.16	429.62	-	317.06, 301.13, 270.21, 169.04	Tamarixetin derivative	[30]

### Nuclear magnetic resonance (NMR) analysis

To provide a broader scope of the *n****-***butanol fraction *T. nilotica* flowers metabolome, ^1^H-NMR was used to provide insights into both secondary and primary metabolites that were not detected by LTQ-LC-MS-MS. ^1^H-NMR can also be used for structural elucidation and determining major metabolites. Sugars, flavonoids, phenolics, and coumarins were among the major metabolites classes detected in the *n*-butanol fraction of *T. nilotica* flowers using ^1^H-NMR as detailed in (Table 3).

**Table 3:** The identified metabolites of the *n*-butanol fraction of *T. nilotica* flowers exhibited at ^1^H-NMR

Functional Groups	^1^H-NMR (m,* J* in Hz)
**M1 Un/saturated fatty acids**	
** 18- CH**_**3**_	0.9
** (CH**_**2**_**)**_**n**_	1.2
** 2-CH**_**2**_	1.6
** 3- CH**_**2**_	2.07
** allylic CH**_**2**_	2.29
** Olefinic CH**	5.33
**Sugars**	
** M2** ***α*-glucose**	5.18 (d, *J* = 3.8 Hz)
** M3** ***β*-Glucose**	4.58 (d, *J* = 7.8 Hz)
** M4 sucrose**	5.40 (d, *J* = 3.8 Hz), 4.17 (d, *J* = 8.5 Hz)
**Organic acids**	
** M5 Succinic acid**	2.56 (s)
**Coumarins & flavonoids**	
**Coumarins derivative**	6.35, 7.60 (d, *J* = 15.8 Hz)
** Flavonoids derivative**	6.2–8.23

**Fig. 4:**
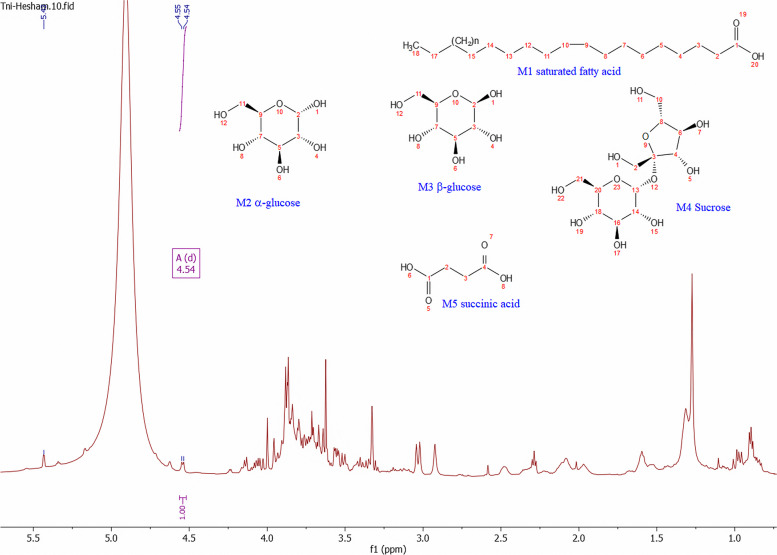
^1^H-NMR spectrum exhibiting the identified metabolites in the *n*-butanol fraction of *T. nilotica* flowers; primary metabolites i.e., fatty acids and sugars (M1-M4) as well as organic acid (M5) at the aliphatic region δ_H_ 0.5—5.5 ppm as mentioned in Table 3

Fatty acids were discriminated against by the presence of terminal (CH_3_ ) at *δ*_H_ 0.9 ppm, long chain methylene groups at *δ*_H_ 1.2 ppm, and olefinic (CH) showed at *δ*_H_ 5.3 ppm, as shown in (Fig. 4, M1).

**Fig. 5:**
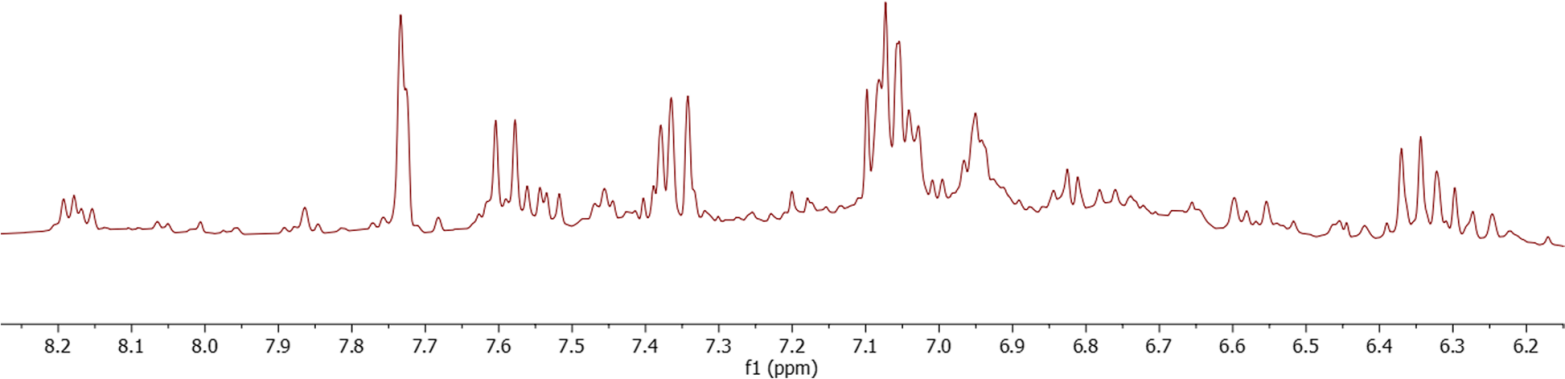
^1^H-NMR spectrum exhibiting the identified metabolites in the *n*-butanol fraction of *T. nilotica* flowers; in aromatic region δ_H_ 5.5—8.2 ppm prescribing coumarins and flavonoids

Sugars, the second intense metabolites, were recognized by the presence of anomeric proton annotated as, α, β glucose, and sucrose, which exhibited anomeric protons at *δ*_H_ 5.18 (d, *J* = 3.8 Hz) for (Fig. 4, M2), *δ*_H_ 4.58 (d, *J* = 7.8 Hz) (Fig. 4, M3), and *δ*_H_ 5.40 (d, *J* = 3.8 Hz), *δ*_H_ 4.17 (d, *J* = 8.5 Hz) (Fig. 4, M4), respectively. Moreover, CHs attached to hydroxyl groups exhibited overlapped peaks at a range of *δ*_H_ 3.2—4.02 ppm as shown in (Fig. 4, M2-M4) [91]. A sharp singlet peak at *δ*_H_ 2.56 (s) indicated the presence of a common organic acid elucidated as succinic acid (Fig. 4, M5) [91]. Finally, flavonoids and coumarins were found in a region of aromaticity, which was recognized by the presence of *δ*_H_ 6.35, 7.60 (d, *J* = 15.8 Hz) corresponding to *α*, *β* unsaturated ketone in coumarins. Concerning flavonoids overlapped peaks at the region of *δ*_H_ 6.0—8.33 ppm, which was elucidated with the help of LTQ-LC-MS-MS data (Fig. 5).

### Cytotoxic evaluation of the *n*-butanol fraction of *T. nilotica* flowers

The cytotoxic effect of the *n*-butanol fraction *T. nilotica* flowers was investigated as a cytotoxicity SRB quick screening against MCF-7 and Huh-7 cells. The *n*-butanol fraction inhibited cancer cells in a dose-dependent manner since the activity increased with increasing the dose. For instance, at a concentration of 100 µg/ml, the viability percentage was 54.27% compared to 100% with 10 µg/mL on MCF-7 with an IC_50_ ˃100 µg/mL. However, the best effect was observed with Huh-7 where the percentage viability decreased from 51.89% at 10 µg/mL to 7.22% at 100 µg/mL with an IC_50_ = 37 µg/mL (Table 4).

**Fig. 6:**
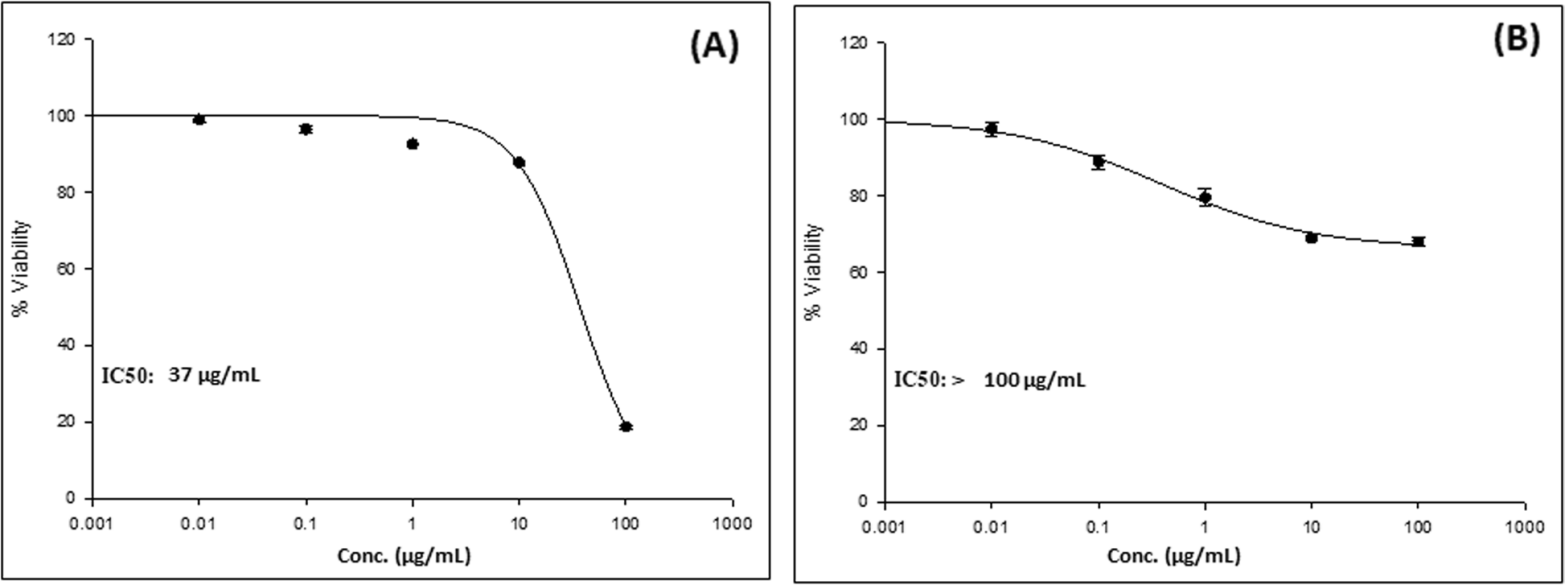
In-vitro SRB cytotoxicity assay of the *n*-butanol fraction of *T. nilotica* flowers against **A**: Huh-7 and **B** MCF-7 cell lines in increasing concentrations (0.01–100 µg/mL). Data points are expressed as mean ± SD (*n* = 3)

Cell viability was assessed at five different concentrations (0.01, 0.1, 1, 10, and 100 µg/mL) using the SRB assay revealed that *T. nilotica* flowers *n*-butanol fraction possesses a dose-dependent cytotoxic effect with an IC_50_ of 37 µg/mL with Huh-7 cell lines while it showed IC_50_ > 100 µg/mL with MCF-7 cell lines (Fig. 6).

**Table 4:** Cytotoxicity SRB quick screening results of the *n*- butanol fraction of *T. nilotica* flowers

Tested sample concentration	Cell viability %	
	Cancer Cell lines	
	Huh-7	MCF-7
**10 µg/mL**	51.89	100
**100 µg/mL**	7.22	54.27

**Fig. 7:**
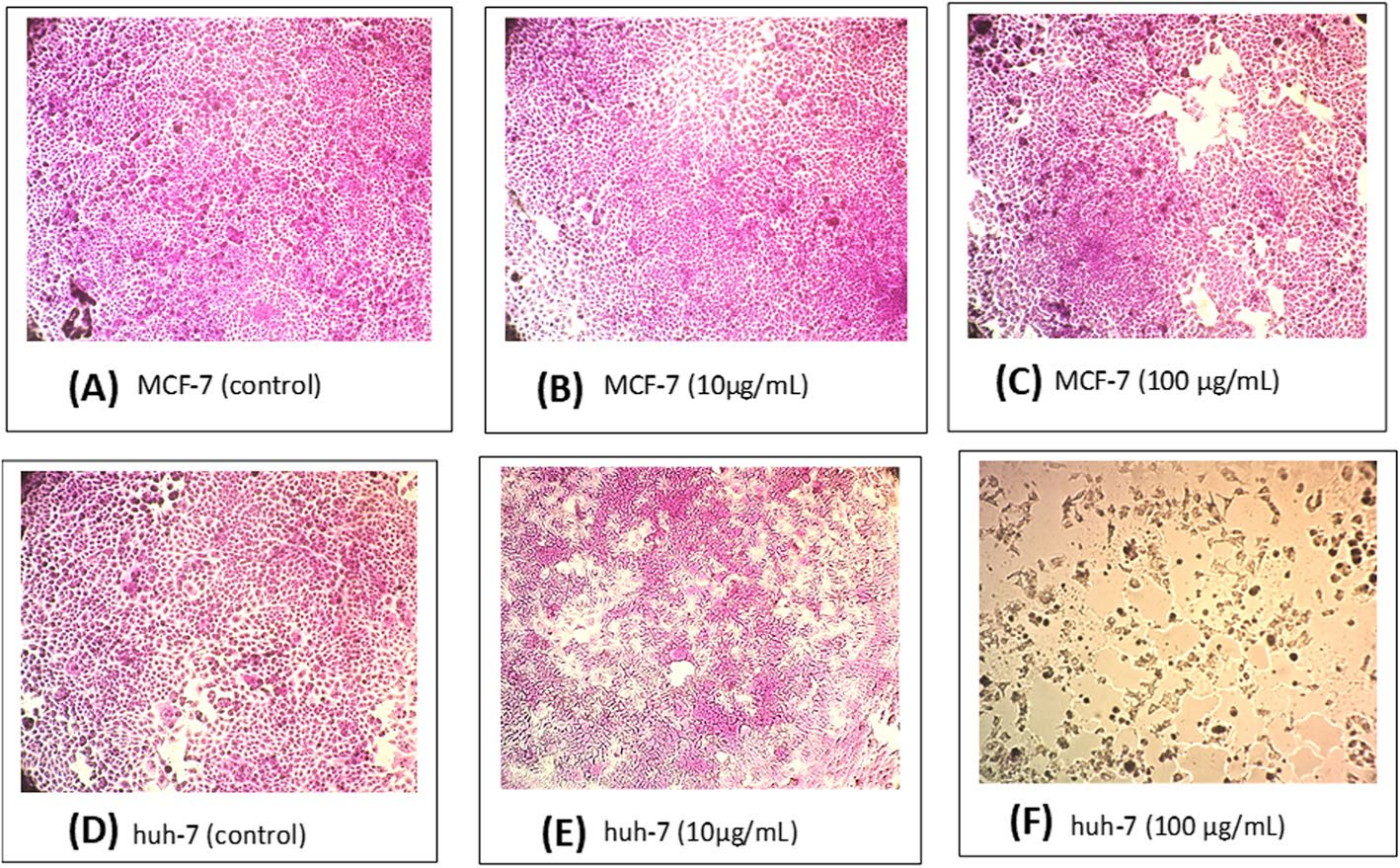
Optical microscope-stained images of quick screening SRB cytotoxicity assay of the *n*- butanol fraction of *T. nilotica* flowers against **MCF-7**; **A:** negative control, **B:** 10 µg/mL, **C:** 100 µg/mL, and **Huh-7**; **D:** negative control, **E:** 10 µg/mL, **F:** 100 µg/mL

## Discussion

One of the leading causes of death on the globe is cancer. Given their significant toxicity to cancer cells, natural products, and their secondary metabolites are highly significant for research into potential anticancer treatments. Previous research found that several *Tamarix* species have displayed varying cytotoxic activities. Breast adenocarcinoma cells (MCF-7) were suppressed by the methanolic extract of *T. aphylla* in a concentration-dependent manner [33]. Different extracts of *T. senegalensis* demonstrated anti-cancer effects in human liver (Huh-7) and lung (A-549) carcinoma cells [31]. *T. gallica* shoots, flowers, and leaves methanolic extracts were able to inhibit the proliferation of colon cancer (Caco-2) cells at concentrations of 50 and 100 g/mL [82]. Furthermore, *T. articulata* methanolic extract demonstrated promising antiproliferative activity against hepatocellular carcinoma [92], as well as against prostate cancer (LnCaP) cells’ motility and invasiveness in a dose-dependent manner [93]. In this study, the *n*-butanol fraction of *T. nilotica* flowers showed cytotoxic activity against MCF-7 and Huh-7 cells (Fig. 6) in a dose-dependent manner with a more promising effect against liver cancer cell Huh-7 (IC_50_ = 37 µg/mL). The optical microscope-stained images were recorded as shown in Fig. 7 comparing the cytotoxic effect of *n*-butanol fraction of *T. nilotica* flowers at a concentration of 10 and 100 µg/mL with comparison to (-ve control). Images clearly show the cytotoxic effect of the extract against MCF-7 and Huh-7 cell lines (Fig. 7C, E & F) where no morphological changes were observed on MCF-7 at conc. 10 µg/mL (Fig. 7B) as well as the negative control of both cell lines (Fig. 7A & D) while more potent effect was observed against Huh-7 (Fig. 7E & F). This confirms that the *n*-butanol fraction of *T. nilotica* flowers possess cytotoxic effects which are clearer and more potent on Huh-7 cells over MCF-7 cells.

*T.nilotica* has been previously reported for promising cytotoxic activity against human colon (HCT-116) and breast (MCF-7) cancer cells [94], whereas ethyl acetate was active against lung cancer cell line with increased expression levels of p-53 and Bax whereas that of Bcl-2 was decreased [36, 37], while flowers were effective and selective against liver cell carcinoma (Huh-7) [38].

The chemical investigation of various *Tamarix* species was reported. Gallic acid, flavones, and flavonols were among the polyphenols found in this study that were recognized as compounds that had previously been found in other species of *Tamarix* [34, 95]. For example, a study on the alcohol-soluble fraction of an aqueous extract of *T. nilotica* aerial parts collected from Egypt and Saudi Arabia was discussed by Sekkien A. et al. 2018 [30]. The study reported that the major compounds in the Egyptian species extract were (iso)ferulic acid-3-sulphate, methyl ferulate sulfate, and coniferyl alcohol sulfate derivative. Moreover, this species exhibited the presence of kaempferide, gallic acid, nilocitin, kaempferol dimethyl ether sulfate, tamarixetin, kaempferol, quercetin, methyl gallate methyl ether, kaempferol 3-*O*-*β*-glucuronide and 4ʹ-O-methyl quercetin 3-*O*-*β*-hexoside which was following the identified compounds in our study [30]. Also, the tannin-identified compounds in our study as hirtellin B, gemin D, nilotinin D1, and tamarixinin A were following those reported in *T. nilotica*, *T. pakistanica*, *T. tetrandra*, and *T. senegalensis* by [56, 64, 68, 96]. These several identified polyphenolic compounds in this genus explain its widespread biological activity as stated in [11].

The phytochemical analysis of the *n*-butanol extract of *T. nilotica* flowers using LC-MS/MS analysis reveals the identification of various phenolic compounds such as gallic acid, caffeic acid, ferulic acid, luteolin, kaempferol, quercetin, kaempferol-3-*O*-glucuronide, tamarixetin, besides various galloyl and gallate moieties. Fragments at *m/z* [M-H-152]^−^ and [M-H-170] ^–^ denoted the losses of galloyl and gallate moieties respectively, eliminated by gallotannins or galloylated esters [60]. Tannins were previously isolated and identified in *T. nilotica* and have shown potent cytotoxic effects with high tumor specificity [68]. The promising cytotoxic effect against liver carcinoma can be well correlated with the tentatively identified phenolic compounds where caffeic and gallic acid was reported to reduce the growth of MCF-7 breast cancer cells and altered the expression of apoptotic genes [97], ferulic acid also promotes apoptosis in cancer cell lines MCF-7 and HepG-2 and activated the caspase-8 and − 9 pathways, has cytotoxic action and [98]. while nilocitin showed a G2/M and S cell cycle arrest as a consequence of the G1 phase [99], furthermore, the flavonoid hispidulin (4’,5,7-trihydroxy-6-methoxyflavone) causes ERS-mediated apoptosis in hepatocellular carcinoma cells by stimulating the AMPK/mTOR pathway, [100]. HepG-2 cells were more vulnerable to hispidulin-mediated cell death than immortalized L929 fibroblasts, indicating that this substance has a distinct level of toxicity in tumor-related cell lines than normal cell lines [101]. When kaempferol was administered to the human breast cancer cell line MCF-7, it suppressed the expression of PLK-1, a protein-like kinase that has been shown to control mitotic development and to be elevated in several human cancers. Kaempferol’s anticancer activity is mediated via inhibition of the EGFR-related Src, ERK1/2, and AKT pathways, and it may be a powerful inhibitor of pancreatic cancer cells [102]. Luteolin is a very significant flavonoid that is present in many foods. It has several health benefits, including its ability to prevent cancer, induce cell cycle arrest and apoptosis in some human cancer cells, and enhance the antitumor effects of 5-FU on Bel7402 and HepG-2 cells. These effects may be connected to apoptosis and the control of 5-FU metabolism [103–105]. The dietary flavonoid quercetin, which is found in berries, demonstrated high cytotoxicity it prevented HepG-2 cancer cells from proliferating and surviving while inducing apoptosis by increasing the expression of p53 and BAX [106, 107].

Our findings imply that the *T. nilotica* flower’s *n*-butanol fraction has the potential to be a promising cytotoxic candidate against Huh-7 cancer cells.

## Conclusion

This study documents a detailed metabolites profiling for the unexplored *n*-butanol fraction of *Tamarix nilotica* flowers. A total of 39 constituents including tannins, flavonoids, and phenolic acids, were tentatively identified. The in vitro cytotoxicity study revealed significant cytotoxic action towards the hepatocyte-derived cellular carcinoma cell lines, human liver (Huh-7). However, further studies are necessary to correlate this activity to the identified compounds to demonstrate *T.nilotica* as a prospective drug candidate that inhibits cancer.

## Data Availability

The datasets generated and analyzed during the current study are all mentioned in the presented manuscript.
